# INTERACTIONS BETWEEN CHEMICAL AND CLIMATE STRESSORS: A ROLE FOR MECHANISTIC TOXICOLOGY IN ASSESSING CLIMATE CHANGE RISKS

**DOI:** 10.1002/etc.2043

**Published:** 2012-12-18

**Authors:** Michael J Hooper, Gerald T Ankley, Daniel A Cristol, Lindley A Maryoung, Pamela D Noyes, Kent E Pinkerton

**Affiliations:** † U.S. Geological Survey, Columbia Environmental Research CenterColumbia, Missouri; ‡ U.S. Environmental Protection Agency, Office of Research and Development, National Health and Environmental Effects Research Laboratory, Mid-Continent Ecology DivisionDuluth, Minnesota; § Institute for Integrative Bird Behavior Studies, Department of Biology, The College of William and MaryWilliamsburg, Virginia, USA; ‖ Department of Environmental Sciences, University of CaliforniaRiverside, California, USA; # Nicholas School of the Environment, Duke UniversityDurham, North Carolina, USA; †† Center for Health and the Environment, University of California at DavisDavis, California, USA

**Keywords:** Adverse outcome pathway, Acclimation, Weather

## Abstract

Incorporation of global climate change (GCC) effects into assessments of chemical risk and injury requires integrated examinations of chemical and nonchemical stressors. Environmental variables altered by GCC (temperature, precipitation, salinity, pH) can influence the toxicokinetics of chemical absorption, distribution, metabolism, and excretion as well as toxicodynamic interactions between chemicals and target molecules. In addition, GCC challenges processes critical for coping with the external environment (water balance, thermoregulation, nutrition, and the immune, endocrine, and neurological systems), leaving organisms sensitive to even slight perturbations by chemicals when pushed to the limits of their physiological tolerance range. In simplest terms, GCC can make organisms more sensitive to chemical stressors, while alternatively, exposure to chemicals can make organisms more sensitive to GCC stressors. One challenge is to identify potential interactions between nonchemical and chemical stressors affecting key physiological processes in an organism. We employed adverse outcome pathways, constructs depicting linkages between mechanism-based molecular initiating events and impacts on individuals or populations, to assess how chemical- and climate-specific variables interact to lead to adverse outcomes. Case examples are presented for prospective scenarios, hypothesizing potential chemical–GCC interactions, and retrospective scenarios, proposing mechanisms for demonstrated chemical–climate interactions in natural populations. Understanding GCC interactions along adverse outcome pathways facilitates extrapolation between species or other levels of organization, development of hypotheses and focal areas for further research, and improved inputs for risk and resource injury assessments. Environ. Toxicol. Chem. 2013;32:32–48. © 2012 SETAC

## INTRODUCTION

A variety of environmental variables influenced by global climate change (GCC) can directly or indirectly affect the health of organisms. These variables include temperature, salinity, pH, and penetration of ultraviolet (UV) radiation in aquatic environments. Global climate change is causing increases in the severity and frequency of droughts and extreme precipitation events, as well as regional-scale declines in air quality (e.g., increased ground-level ozone and particulate matter) in terrestrial systems 1, 2. Direct effects of these GCC-related changes have been and continue to be characterized in biota, for example, the potential influence of temperature on distributions of fish populations 3–6 and the timing of avian migrations to nesting grounds and their concordance with appropriate prey availability 7–9. However, far less is known about the indirect effects of variables affected by GCC on humans and the environment, including the potential for interactions with toxic chemicals. Factors such as temperature can greatly influence the toxicity of chemicals in a variety of taxa 10, 11. Other than for a few species, chemicals, and endpoints, however, data collected to date concerning the effects of changes in the global climate on the toxicity of chemicals are not comprehensive enough to routinely support integrated risk assessments. Hence, there is a need to develop approaches and tools to better enable consideration of potential interactions of toxic substances with factors affected by GCC.

Risk assessments for toxic chemicals historically have relied on apical, whole-organism end points directly related to key demographic processes such as survival, growth, and reproduction. Data reflecting mechanistic aspects of biological effects of toxic chemicals—such as altered gene or protein expressions, metabolite profiles, and histopathology—typically have received little or no direct use in either human or ecological risk assessment. Yet, mechanistic information can help to address fundamental uncertainties inherent to current risk-assessment approaches, including those related to chemical effect extrapolations across species, endpoints and chemical structures, as well as variations in risk outcomes across settings 12, 13. Importantly, understanding the biological pathways through which chemicals exert their effects provides a means of assessing the impacts of mixtures with other chemicals, as well as interactions of chemicals with nonchemical stressors. This is directly relevant to evaluating potential interactions between toxic chemicals and environmental conditions influenced by GCC. Because it is impractical, if not impossible, to collect empirical data concerning all interactions between environmental variables affected by GCC and chemicals in the environment (including toxicants and toxins of concern as well as chemicals lacking classification as contaminants), it is necessary to develop predictive approaches to help assess where, when, and how these interactions might influence toxicity at organismal and higher levels of organization and conclusions concerning potential risk. To support these types of predictive approaches, it is necessary to incorporate mechanistic data into the risk-assessment process for evaluation of the potential influence of GCC on chemical toxicity.

A major impediment to the use of mechanistic data in risk assessments has been an inability to clearly translate information collected at lower levels of biological organization (e.g., molecular, biochemical, and cellular responses) into endpoint alterations that are meaningful to risk assessment, namely, effects on individuals and populations. To address this limitation, Ankley et al. 14 described a conceptual framework based on constructs called adverse outcome pathways (AOPs), which depict linkages between molecular initiating events (interaction of chemicals with biological targets) and the subsequent cascade of responses that occur across biological levels of organization that culminate in impacts on individuals (or populations) that can be used for assessing risk. The basic concepts underpinning AOPs are not new—variations on the theme have been captured to different degrees through constructs like mechanisms or modes of action and/or toxicity pathways 14. For example, the National Research Council has recently proposed toxicity testing regimes centered on evaluations of biological perturbations along key toxicity pathways using methods in computational biology and in vitro tests based on human biology 15. What the AOP concept provides is a unified framework based on defining relevant linkages across biological levels of organization in the context of applied risk assessment. Since initial description of the AOP framework, subsequent efforts have focused on further development and application of the concept, including a Pellston conference sponsored by the Society of Environmental Toxicology and Chemistry in 2009 that specifically addressed topics related to the derivation of AOPs from existing and new data, incorporation of population modeling into the framework, and the use of AOPs to better understand the resilience of systems and extrapolation of chemical effects across species 16–20.

**Fig. 1:**
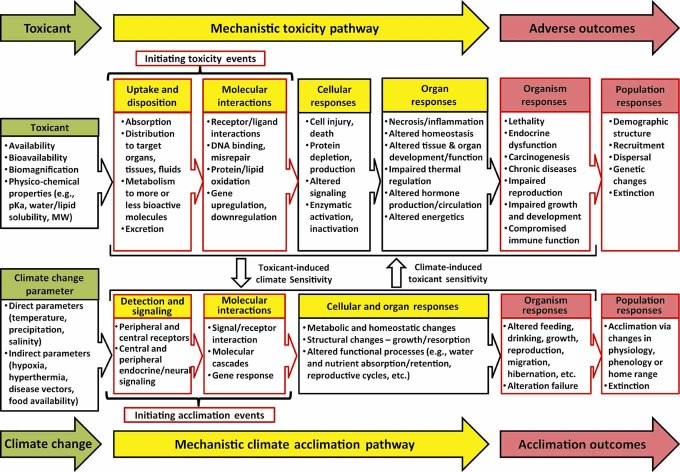
Adverse outcome and climate acclimation pathways suggested for mechanistic assessments of contaminants and global climate change interactions. Modified from Ankley et al. 14. [Color figure can be seen in the online version of this article, available at http://wileyonlinelibrary.com.]

In the present analysis, we describe adaptations of the AOP concept and by extension mechanistic data as tools to help identify potential adverse effects elicited by GCC and chemical toxicant exposures. The ultimate goal of this effort is to develop assessment approaches and tools that enable predictions of when and how significant interactions between chemical and nonchemical stressors might be expected to occur, rather than solely relying on collections of empirical data for different combinations of stressors. Figure 1 shows a modification of the AOP framework as originally depicted 14. Notable modifications include incorporation of an exposure component that could be affected by GCC variables influencing chemical fate, transport, and bioavailability and consideration of internal dosimetry (toxicokinetics), which also could be affected by, for example, changes in temperature in poikilotherms 10. In considering this framework, it becomes apparent that there are different relevant ways in which variables affected by GCC could interact with chemicals in terms of producing adverse effects.

One important dichotomy involves toxicant-induced changes that alter the ability of an organism to respond to GCC stressors (toxicant-induced climate susceptibility [TICS]), in contrast to climate-induced toxicant sensitivities (CITS), the scenario where GCC affects the toxicity of chemicals. Such CITS scenarios produce altered or enhanced toxicity of chemicals under climate change conditions that might not be predicted in controlled laboratory testing or among natural populations residing within or at the edge of their physiological tolerance ranges. The former scenario (TICS) involves alterations caused by toxic chemicals that impact the ability of an organism to acclimate to a stress introduced by GCC through various biochemical, physiological, or behavioral responses. These responses can be metabolic (hyperphagia, shivering, estivation, and hibernation), structural (metamorphosis, tissue resorption and growth), or homeostatic (water retention, ion balance, nutrient absorption, and reproductive processes), and can trigger associated behavioral changes (feeding, drinking, reproduction, and migration; Fig. 1). Successful acclimation allows survival in the face of environmental change, while failure or environmental change beyond the ability of an organism to acclimate can lead to loss of individuals or local populations or even to species extirpation or extinction 21. Neither contaminant effects on acclimation processes nor climate effects on contaminant toxicity may be obvious in laboratory studies carried out under carefully controlled conditions that reduce testing variability and protect test animals from all stressors but the test chemical.

In the present study, we present a number of real-world examples illustrating how an understanding of mechanistic toxicology supports integration of known or hypothesized interactions between chemical and nonchemical/GCC stressors into chemical risk assessments. We include examples relevant primarily to ecological health and demonstrate how the framework shown in Figure 1 can be applied to either prospective or retrospective risk assessments. Specifically, we present several scenarios where, based on established or plausible interactions between chemical and GCC-related nonchemical stressors, prospective assessments of risk can be developed that help to identify potential adverse effects between existing or new chemical exposure patterns and GCC stressors that have yet to be investigated or fully documented. We also describe retrospective situations in which observed impacts in humans and wildlife can be dissected and diagnosed relative to toxic pathways and mechanistic interactions between chemical and nonchemical stressors. An understanding of toxic pathways in one population or species can then serve as a basis for developing predictive models that can be applied in a generalized manner, for example, at sites or in situations removed from where initial observations were made.

As GCC–chemical interactions might occur all along mechanistic toxicity pathways, case studies depict examples focusing first on exposure and the disposition of toxicants, followed by examples depicting toxicodynamic interactions between chemicals, effect-associated receptors, and GCC. More complex scenarios then demonstrate the interplay between CITS and TICS and finally illustrate how retrospective analysis using AOPs can help to explain observed GCC-associated toxicity lacking a known cause–effect relationship.

## ULTRAVIOLET-INDUCED PHOTOACTIVATION OF POLYCYCLIC AROMATIC HYDROCARBONS

**Fig. 2:**
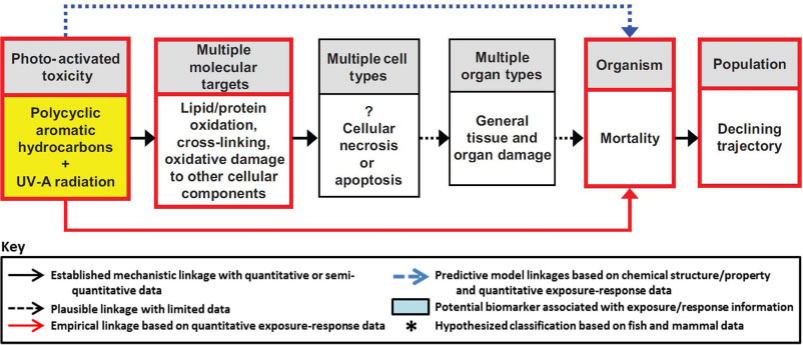
Adverse outcome pathway of the interaction of ultraviolet radiation with polycyclic aromatic hydrocarbons. With permission from Ankley et al. 14. [Color figure can be seen in the online version of this article, available at http://wileyonlinelibrary.com.]

One manner through which environmental variables influenced by GCC could affect contaminant toxicity involves direct effects of the variable on chemical characteristics. The polycyclic aromatic hydrocarbons (PAHs) are a class of compounds whose environmental concentrations, through pyrogenic events such as forest fires, are projected to increase as a consequence of GCC 22. The toxicity of PAHs can occur via a variety of molecular initiating events and pathways. A PAH AOP of particular relevance to GCC involves activation to reactive species by ultraviolet (UV) radiation in sunlight, a process termed photoactivated toxicity (PAT; Fig. 2). The intensity of UV radiation, a key factor in determining PAH PAT, is likely to be affected by variables altered by GCC 1, 2. These variables could include decreases in pH that can clarify water, thereby increasing exposure of aquatic animals to UV radiation, and increased inputs of dissolved or particulate organic carbon to aquatic systems, which would effectively reduce UV penetration. Hence, specific influences of GCC on UV intensity in aquatic systems are likely to be site- and situation-specific. However, through understanding the mechanisms by which PAT occurs, it is possible to apply predictive models to the assessment of the relative risk of PAH toxicity under GCC.

Almost 30 years ago researchers noted that toxicity of the PAH anthracene to fish could be increased by an order of magnitude or more through simultaneous exposure to sunlight 23, and a wide array of studies since that time have documented similar effects with other PAHs in many different aquatic species (for reviews, see 24–26). Ankley et al. 25 provide an overview of different aspects of mechanisms of PAH PAT and describe the derivation of an AOP specific for this phenomenon, focusing on lethality in aquatic species (Fig. 2) 14. Here, we briefly describe the pathway, with a particular emphasis on mechanism-based models that enable prediction of risk under scenarios of varying UV intensity and PAH concentrations, such as those that could occur as a consequence of GCC. The initial step in PAT is uptake of PAHs by sensitive species such as larval fish or pelagic invertebrates. Once in the animal, some PAH structures can be activated through interactions with UV radiation in sunlight, typically in the UVA portion of the spectrum (320–400 nm). Models have been developed that enable prediction of those PAHs likely to exhibit PAT based on comparatively easily measured or calculated structural characteristics 27. The interaction of UV radiation with accumulated PAHs involves elevation of the ground-state molecule to excited singlet states, which can release excess energy through a variety of mechanisms, including decay to relatively longer-lived, triplet-state molecules capable of interacting with molecular oxygen to form reactive singlet oxygen. Singlet oxygen can interact with a wide variety of biological macromolecules (e.g., proteins, lipids), producing the damage that results in PAT, which typically is manifested, at least in short-term exposures, as lethality 25. Critically, the occurrence of PAT can be accurately modeled or predicted as a function of the product of (internal) PAH dose and UV exposure 28. Variations on this very basic UV × PAH relationship can be incorporated into models such that the PAT of mixtures of PAHs with varying phototoxic potency also can be predicted 29.

Our example concerning PAT has several noteworthy characteristics from the standpoint of assessment of interactions between chemicals and variables influenced by GCC. First, this example clearly shows how information and models derived from knowledge of toxicity mechanisms could be used to make predictions of risk under changing environmental conditions. Both key determinants of toxicity, PAH exposure and UV intensity, could vary as a function of GCC, likely in a very site-specific manner, so it is critical to have tools capable of simultaneously assessing the possible consequences of alterations in either or both parameters. Based on past research, enough is known about the physiochemical and biological mechanisms of PAT that robust existing models could be utilized for the purpose of assessing different risk scenarios associated with GCC. This example also provides an illustration of a possible adverse response that in some situations could well be decreased by GCC. Specifically, in situations where PAH exposure is unchanged but UV penetration in aquatic systems decreases due to increased precipitation and surface runoff, the potential for PAT decreases.

## TEMPERATURE AND SALINITY IMPACTS ON CHEMICAL DISPOSITION

Beyond the accessibility or availability of chemical and nonchemical stressors to organisms, environmental alterations with GCC have the potential to alter the biological disposition (or toxicokinetics) of chemicals that can in turn influence internal exposure sites, concentrations, and durations. Toxicokinetic modifications can lead to changes in organism-level responses and eventually produce population-level impacts. In our model AOP, this would be captured by alterations in absorption, distribution, metabolism, and excretion (ADME) of toxicants (Fig. 1). Understanding how ADME is impacted by different GCC stressors, such as temperature and salinity in aquatic environments, would help to identify mechanisms involved with CITS and allow risk assessors to make predictions about geographical regions susceptible to these types of interactions and other combinations of climatic changes and toxicant exposures.

Some broad observations can be made regarding potential climate effects on the uptake and disposition of chemical contaminants 10. For example, it has generally been observed that uptake and elimination of toxicants increase as temperature increases. Literature on the distribution of toxicants as impacted by GCC factors is limited, and thus few general trends have been described. Increases in two GCC-related variables, temperature and salinity, have been shown to enhance metabolism to typically more, but in some cases less, toxic metabolites (see Noyes et al. 10 for discussion). Two examples using the AOP framework to illustrate how ADME mechanisms interact with GCC stressors and lead to interactive effects are (1) alterations in polychlorinated biphenyl (PCB) metabolism with increasing temperatures, and (2) alterations of pesticide toxicity with changes in temperature and salinity.

### PCB uptake and disposition with rising ambient temperatures

Persistent organic pollutants, such as PCBs, are problematic in that they can migrate thousands of miles from their original point of release to high-latitude ecosystems. For example, the Mackenzie River basin in northwestern Canada has been experiencing substantial warming trends and declining snow cover. While atmospheric concentrations of PCBs have generally declined in the Arctic with elimination and reductions in use, increasing trends of contamination by more highly chlorinated PCBs have been measured in Mackenzie River sediments and the predatory arctic fish burbot (*Lota lota*) over a 30-year collection period 30. Study evidence suggests that increasing primary production of algae from rising temperature and declines in snow cover may increase partitioning of PCBs from the water column, leading to increased contaminant bioavailability and transfer up the food chain.

Increasing trends of PCBs in biota are important because environmental conditions being altered by GCC can also influence the biological disposition of these compounds. Several studies have shown that fish metabolize PCBs by cytochrome P-450 (CYP) mixed-function oxidases to more toxic, hydroxylated PCBs (OH-PCBs). For example, the role of temperature in the disposition of PCBs has been examined in juvenile rainbow trout (*Oncorhynchus mykiss*) receiving dietary exposures 31. Increasing water temperatures have been shown to decrease the biological half-life of some of the highly persistent PCB congeners in trout. However, increasing water temperatures have also been shown to increase the metabolism of parent PCBs to OH-PCBs as measured in trout plasma. Several studies have demonstrated different mechanisms of OH-PCB toxicity, including that OH-PCBs can act as agonists for estrogen receptors 32 and displace thyroid hormone from plasma transport proteins 33. Thus, an AOP approach could be used prospectively in this situation to identify important mechanistic toxicity pathways and teleost populations potentially at risk to the dual stresses of increasing temperatures under GCC and PCB contamination.

### Pesticide uptake and disposition with rising temperatures

Effects of temperature on pesticide toxicity can be demonstrated by contrasting the toxicity of organophosphorus insecticides (OPs) and pyrethroids in the midge (*Chironomus dilutus*, formerly *Chironomus tentans*). In these poikilothermic organisms, increased chemical uptake and enhanced metabolic transformation to more toxic oxon metabolites generally accompany increased temperatures. These adverse interactions have been demonstrated, for example, in 96-h midge lethality studies with methyl parathion and chlorpyrifos, where acute toxicity increased as temperatures increased from 10 to 30°C due to increased uptake and likely increased metabolic activation of the parent OPs to their more toxic oxon forms 34. This metabolic activation is necessary for OP toxicity not only in midges but in most organisms, because acetylcholinesterase, the molecular target of OPs, is generally orders of magnitude more sensitive to the oxon moiety. Because of increased activation rates, GCC-induced temperature elevations will likely lead to increased toxicity of OPs to any poikilotherms. As temperature-dependent hydrolysis rates of OPs will likely increase in warming aquatic environments, elevated toxicity may be tempered by decreased persistence of toxic OP concentrations.

Alternatively, type 1 and some type 2 pyrethroids demonstrate decreased toxicity at elevated temperatures in poikilotherms, including the midge. Sensitivity of the midge to permethrin and lambda-cyhalothrin has been shown to decrease with increasing temperatures 35. Two factors that contributed to the inverse relationship between the toxicity of pyrethroids and temperature included decreased neuronal sensitivity and increased metabolism of parent compounds at elevated temperatures. Contrary to the requirement for metabolic activation of OPs, parent pyrethroids are the toxic form of these pesticides, while their hydrolyzed metabolites are readily excreted. Decreased metabolic rates at lower temperatures were shown to decrease hydrolysis and elimination rates of pyrethroids, maintaining the toxic form of the pesticide in the neuron, leading to greater toxicity.

Conversely, some type 2 pyrethroids have demonstrated increasing toxicity with higher ambient temperatures, including in cockroaches (*Blattella germanica*) 36, water fleas (*Daphnia magna*) 37, leopard frogs (*Rana* sp.) 38, and grass shrimp (*Palemonetes* sp.) 39. The α-cyano moiety contained on type 2 pyrethroids may be responsible for imparting greater toxicity at higher temperatures among some organisms, although the mechanism is not understood. Thus, elevated temperatures under GCC could increase or decrease the toxicity of pyrethroid insecticides depending on the species and specific pyrethroid exposure, demonstrating the complexity of these interactions and continued gaps in our understanding of pyrethroid toxicity mechanisms.

### Uptake and disposition of pesticides with altered salinity

Sea-level rise linked to thermal expansions, reduced snow cover, and the accelerated melting of glaciers, ice caps, and polar ice sheets is projected to increase the salinity of estuarine and coastal freshwater habitats 2. For example, regions with diminished snow-pack runoff due to GCC are likely to experience increases in estuarine salinity. Projected temperature increases of 2.1°C by 2090 are forecasted to result in a loss of approximately half the average April snow-pack storage for the San Francisco Bay estuary region. A reduction of approximately 20% of historical annual spring runoff would cause increases in salinity of up to 9 psu (∼9 g/L) in select regions 40. Estuarine areas, such as the San Francisco Bay, may be especially susceptible to GCC and toxicant interactions due to the diversity of species present, some of which are endangered (e.g., delta smelt [*Hypomesus transpacificus*]), and the fact that these areas are increasingly impacted by anthropogenic activities, including substantial point and nonpoint discharges of chemicals into the system.

The relevance of increased salinity on the toxicity of chemicals has been demonstrated in coho salmon (*Oncorhynchus kisutch*) acclimated to different salinity conditions and subsequently exposed to the OP phorate in 96-h acute toxicity tests. These fish demonstrated a 30-fold increase in acute toxicity to phorate when acclimated to 32 g/L salinity, compared with those acclimated at <0.5 g/L salinity 41. The present study also demonstrated an increase in formation rates of the toxic phorate oxon and highly toxic phorate oxon sulfoxide metabolites in liver, gill, and olfactory microsomes under the 32-g/L salinity regime. This and other studies (with aldicarb and fenthion, below) have posited that the increased toxicity may be related to the differential expression or augmented activity of flavin-containing mono-oxygenases (FMOs), which are involved in the osmoregulation and metabolism of xenobiotics (e.g., OP activation by oxidation of peripheral thioether constituents prior to oxon formation). The enhanced activity of FMOs has been shown to also increase toxic metabolite formation of the carbamate pesticide aldicarb, which shares a similar thioether to phorate and acts by acetylcholinesterase inhibition. Exposures of rainbow trout (*O. mykiss*) to aldicarb and elevated salinity increased acetylcholinesterase inhibition, toxicity of the pesticide, and microsomal production of the aldicarb sulfoxide 42. This altered biotransformation of aldicarb was concomitant with enhanced catalytic activity of FMOs and the upregulation in mRNA expression of genes encoding FMOs. In hybrid striped bass (*Morone saxatilis* × *chrysops*), a species that expresses FMOs that are nonresponsive to salinity induction, salinity did not affect acetylcholinesterase activity, toxicity, or aldicarb sulfoxide formation. Coincubations of microsomes with competitive CYP inhibitors had no impact on sulfoxide metabolite formation, suggesting that CYPs were not contributing to the salinity-induced sulfoxide formation. Extension of these findings to assessments of other anticholinesterase compounds that require thioether oxidation (e.g., fenthion) has demonstrated similar increases in toxicity with increases in salinity 43, 44, suggesting that this model could apply to other compounds that are partially metabolized by FMO pathways.

Increased acute toxicity of pesticides (lower median lethal concentration values) resulting from increased temperature and salinity conditions illustrate an AOP whose initiating event, metabolic activation enhanced by climate-modified temperature or salinity conditions, ends with increased lethality at the organism level. Prospective scenarios might suggest that other documented effects of pesticide toxicity in fish, such as interference with olfaction or behavior 45, may be another adverse consequence of these pesticide–climate interactions. For example, salmonids use olfaction to detect chemical cues to provide crucial information about food, predators, reproductive status of mates, environmental contamination, and imprinted characteristics of natal streams. Impairment of this system can have detrimental effects on individuals and populations 46. Thus, understanding the ramifications of GCC on contaminant uptake and disposition can allow risk assessors to anticipate a range of lethal and sublethal responses to contaminants.

In both ADME alteration scenarios (i.e., PCBs and pesticides), interactions occur due to CITS scenarios. The idea of using a prospective approach is also highlighted in both ADME examples because the underlying mechanism is fairly well understood. It may be feasible for risk assessors to make similar types of predictions for other interactions between GCC stressors and other toxicants.

## ENDOCRINE-DISRUPTING COMPOUNDS AND GCC INTERACTIONS

In addition to alterations in chemical uptake and disposition by climate-associated ecosystem changes, the endocrine systems of biota are important targets of chemical toxicants. Because hormone systems are highly integrated into the life functions of organisms, toxicant disruptions of these systems can lead to many specific and nonspecific responses depending on the life stage and condition of the organism at the time of exposure. The vertebrate endocrine system is also highly sensitive to environmental cues, such as precipitation, temperature, and food availability, all of which are being altered by GCC. The combined role of the endocrine system in maintaining internal homeostasis and responding to the external environment makes it a sensitive and important target of toxicant and GCC interactions. The following three case studies provide examples to demonstrate some of these endocrine disruptor and GCC interactions, with a focus on potential perturbations of the thyroid and gonadal endocrine systems.

### Amphibian metamorphosis, GCC, and the hypothalamic–pituitary–thyroid system

Thyroid system functioning can be impaired by exposures to numerous environmental contaminants, including PCBs, polybrominated diphenyl ether (PBDE) flame retardants, PAHs, organochlorine and OP pesticides, metals, sex steroids, and pharmaceuticals. Exposures to thyroid-disrupting chemicals may impair the ability of vertebrates to adequately respond to GCC (i.e., a TICS scenario).

Exposures to thyroid-disrupting chemicals in aquatic breeding amphibians are of special concern because metamorphic transitioning from water-based tadpoles to semiaquatic or terrestrial juveniles depends primarily on programmed secretions of thyroid hormones. Water availability and temperature are key variables affecting the timing of larval transitioning, with evidence of accelerated metamorphosis in amphibian tadpoles subjected to drying conditions and elevated water temperatures 47, 48. This accelerated metamorphosis is considered to be an acclimative mechanism for coping with water- or temperature-stressed environments. Exposures to thyroid disruptors at these types of critical life stages may impact the adaptive capacity of amphibian populations to changing environmental conditions linked to GCC. Thus, early life stages of amphibians may be particularly sensitive to the dual stresses of thyroid-perturbing contaminants and GCC because their thyroid systems are incompletely formed but nonetheless crucial to development.

Thyroid hormones are involved in many important biological processes, particularly those mediating growth, development, reproduction, and metabolism. A substantial body of evidence has identified toxicological mechanisms by which contaminants can disrupt thyroid regulation and thyroid-dependent physiological processes. Several informative reviews have been undertaken that may serve as a reference 49–53. Much of the research that has examined thyroid contaminant effects on the amphibian thyroid system has focused on laboratory measures of their potential to inhibit metamorphosis and damage the thyroid gland 54–59. Little is known about the extent to which thyroid disruptors are affecting wild populations, particularly under important climate change scenarios of elevated temperatures, water shortages, and drought conditions. Also, there continues to be only limited work in amphibians to elucidate toxicological mechanisms of action. However, the thyroid system is generally highly conserved across vertebrates, so mechanistic research in other animal models can help to inform our understanding of effects in amphibians as they relate to potential GCC and thyroid contaminant interactions.

**Fig. 3:**
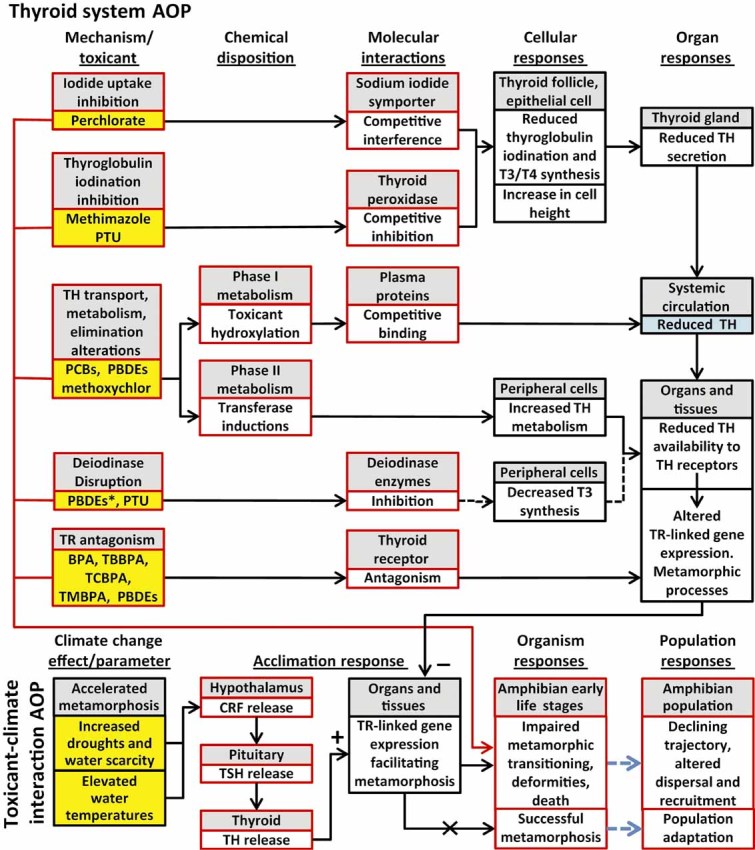
Illustrative adverse outcome pathways (AOP) of toxicant-induced climate sensitivities among amphibians reflecting potential dual interactions between global climate change (GCC) and thyroid-disrupting chemicals (TDCs). Five mechanisms of action are depicted with unique molecular initiating events that have been shown to intersect with reduced thyroid hormone levels and impaired metamorphosis among amphibians. These TDC AOPs share a common outcome that could impair accelerated metamorphosis under GCC. See Figure 2 for key to interactions. BPA = bisphenol A; PBDE = polybrominated diphenyl ether; PCB = polychlorinated biphenyl; PTU = propylthiouracil; TBBPA = tetrabromobisphenol A; TCBPA = tetrachlorobisphenol A; TMBPA = tetramethylbisphenol A; TH = thyroid hormone; T3 = triiodothyronine; T4 = thyroxine; TR = thyroid receptor; CRF = corticotropin-releasing factor; TSH = thyroid stimulating hormone. [Color figure can be seen in the online version of this article, available at http://wileyonlinelibrary.com.]

Specifically, some of the increasingly well-understood molecular initiating events associated with thyroid-toxic contaminant exposures provide an opportunity to use an AOP approach in a prospective analysis to formulate hypotheses to determine and test whether thyroid toxicants could alter organism resilience to GCC-linked water shortages and temperature elevations. Figure 3 provides a representative AOP that outlines the potential interactive impacts of GCC and thyroid disruptor exposures on amphibian metamorphosis. The AOP depicts several molecular pathways that may converge on reductions in levels of circulating thyroid hormones, which are necessary to induce metamorphic transitioning in amphibians.

The biosynthesis and regulation of thyroid hormones (e.g., thyroxine and 3,3′,5-triiodothyronine) are under negative feedback control by the central hypothalamic–pituitary–thyroid axis. Contaminants can perturb the thyroid system at a variety of points along the hypothalamic–pituitary–thyroid axis. Perchlorate, a propellant used by the military, aerospace, and industrial sectors, is a well-documented thyroid disruptor that impairs iodide uptake by the sodium iodide symporter of thyroid epithelial cells 60. Other well-described chemicals that act on the thyroid are methimazole and 6-propyl-2-thiouracil, which are pharmaceutical agents commonly used to treat hyperthyroidism and in thyroid toxicity research as model hypothalamic–pituitary–thyroid axis disruptors. These compounds act on the thyroid by inhibiting thyroperoxidase activity, thereby blocking iodide binding to thyroglobulin and reducing thyroid hormone levels 61.

A variety of compounds have also been shown to alter the transport, metabolism, and elimination of thyroid hormones. For example, OH-PCBs and OH-PBDEs are biotransformation products of phase I metabolism that have been shown to impair thyroid hormone homeostasis by competitively binding to plasma thyroid hormone transport proteins 62–64. In addition, PCBs and PBDEs, among other chemicals, have been shown to impair thyroid hormone homeostasis by enhancing the expression and activity of phase II thyroid hormone conjugating enzymes, such as uridinediphospate glucuronosyl transferases and sulfotransferases, thereby reducing thyroid hormone levels by increasing their catabolism 65–67. Studies in fish have also shown that PBDEs can inhibit the activity of deiodinase enzymes, which are responsible for activating and inactivating thyroid hormones in peripheral tissues 68. The PBDEs and other flame retardants, including tetrabromobisphenol A and tetrachlorobisphenol A, as well as bisphenol A, used in plastics may also disrupt thyroid activity by acting as antagonists to thyroid receptors and altering the expression of receptor-responsive genes 69–71.

The AOP construct in Figure 3 allows for the linkage of several molecular initiating events associated with thyroid contaminants to adverse outcomes at biological levels of organization that are important in risk assessments with special consideration of expected GCC outcomes. In this particular example, the interactions between thyroid disruption and GCC have fairly well-defined biological responses that link several molecular initiating events to an adverse outcome, namely, impaired accelerated metamorphosis in amphibians that might be subjected to GCC-linked water shortages or temperature increases. This type of approach can be very beneficial in prospective analyses of GCC–toxicant interactions because it can serve as the basis for identifying and testing natural populations, with their species-specific life-history characteristics, for these types of complex interactive effects. It can also be used to identify areas where further research is needed and may serve as a model to identify other complex GCC–thyroid contaminant interactions that are potentially problematic.

For example, although not represented in the AOP, plasticity in metamorphic timing has important effects on overall fitness. Amphibian tadpoles that undergo metamorphosis earlier in development as an acclimative response to GCC may bear a fitness cost associated with this rapid metamorphosis in the form of smaller juvenile size. This smaller size has been shown to confer increased sensitivity to predation and reduced fecundity at first reproduction 72–74. Exposure to thyroid disruptors (and other chemical pollutants) could have more deleterious effects on these less fit animals, along with other climate change costressors, such as altered pathogen and parasite distributions 75, 76.

### Fish reproduction and development, GCC, and the hypothalamic–pituitary–gonadal system

Gonadal hormone systems, responsive to both environmental cues and contaminant perturbations, provide examples of how chemical and nonchemical stressors can combine to impact reproductive performance and success, highly relevant organismal and population-level adverse outcomes. The hypothalamic–pituitary–gonadal (HPG) axis controls virtually all aspects of reproduction and sexual development in vertebrates. Normal function of the HPG axis is modulated by a variety of external stimuli such as food availability, photoperiod, behavioral interactions, and, particularly in poikilotherms such as fish, temperature 77. The natural life history of fish species has evolved around these environmental cues, most notably photoperiod and temperature, to optimize cycles of reproduction and development. Some fish species can reproduce and thrive under a fairly broad range of temperatures, while a change in temperature of even a degree or two can completely alter reproductive timing and success in others. As a consequence, increases in temperature associated with GCC have affected and/or are expected to directly affect the abundance and distribution of fish through factors such as alterations in reproductive cycles 3–6, 78–81.

Over the past decade, a substantial amount of research has focused on environmental contaminants, including a number of pesticides, drugs, and industrial chemicals, which have been shown to affect reproduction and development through interactions with the HPG axis. Large-scale screening programs in several countries, including the United States, are designed specifically to identify chemicals that affect HPG functioning through various mechanisms and pathways 82. Although much of the work in this area initially focused on estrogen receptor agonists, there has been increasing concern for and emphasis on other pathways within the HPG axis, including those involving the androgen receptor and synthesis of sex steroids. A wide array of lab and field studies with fish has shown that reproduction and/or development can be adversely affected by contaminants that target any number of pathways within the HPG axis, sometimes at exceedingly low concentrations. For example, a multiyear, whole-lake study with the drug ethinylestradiol demonstrated complete extirpation of the extant fathead minnow population at water concentrations of the synthetic estrogen on the order of 5 to 6 ng/L, which is within the range reported to occur in some municipal effluents 83.

Given that a variety of environmentally relevant chemicals affect HPG functioning in fish and that the HPG axis is controlled, in part, by temperature, it is highly plausible that there would be biologically meaningful interactions between the two types of stressors. Unfortunately, little experimental work has been done to systematically explore this hypothesis. Studies have documented that temperature can affect the induction of vitellogenin (egg yolk protein) by exogenous estrogens in several different fish species 84–87; but other than this one pathway and endpoint, virtually nothing is known about chemical–temperature interactions on HPG function. To assess the scope of potential risk associated with these interactions on HPG functioning in fish, multiple HPG pathways and end points need to be assessed. Without this type of baseline data, it is difficult to speculate exactly what the nature of chemical–temperature interactions might be in terms of decreased fitness. While it clearly is impossible to conduct complex multistressor studies with the many (perhaps thousands) of substances of potential concern from an endocrine perspective, it is possible to generate the needed data in a focused, resource-efficient manner.

Specifically, data collection relative to chemical–temperature interactions can be guided by knowledge of relevant molecular targets and associated AOPs in the fish HPG axis. A large amount of work has been done with HPG-active chemicals in fish, and AOPs have been described for reproductive effects of estrogen receptor agonists and inhibitors of vitellogenesis in females 14. Additional AOPs for the fish HPG axis are being developed for agonists and antagonists of the androgen receptor, inhibitors of key CYP-based enzymes and hydroxysteroid dehydrogenases involved in steroid synthesis, and signaling mechanisms in the brain and pituitary 88. Once a library of high-priority AOPs (probably on the order of 10–20) is assembled, it will be possible to selectively test model chemicals representing these pathways under temperature regimes designed to mimic expected and possible alterations occurring in the environment with GCC. The work ideally would be done using partial and full life-cycle tests with at least two well-established model fish species that utilize different reproductive strategies, for example, continual versus annual spawners. Presumably, some pathways would be more likely to be impacted by temperature interactions than others. In any case, this approach would provide a pathway-specific knowledge base that could be cross-referenced with the mechanism-screening data collected in conjunction with regulatory programs, to identify those HPG-active chemicals that should receive additional scrutiny from the standpoint of potential GCC interactions.

### Hypoxia, GCC, dioxins, and dioxin-like contaminants

Many aquatic environments are subjected to widespread hypoxia (i.e., low dissolved oxygen [DO]) and anoxia (i.e., absence of DO). It is an increasingly urgent global problem that has caused species declines and major ecosystem changes. Hypoxia has been reported across more than 400 aquatic systems, covering thousands of square kilometers, including expansive areas of the Baltic Sea, Gulf of Mexico, and Chesapeake Bay 89–91. The frequency, duration, and geographical extent of hypoxia have increased over the past few decades due to anthropogenic sources of eutrophication from nutrient pollution and fossil fuel burning coupled with wetland losses, increased fertilizer usage, and urbanization 92.

Climate change is projected to worsen hypoxia by increasing nutrient runoff in regions subjected to increasing and extreme rainfall and by increasing oceanic oxygen stratification and warming 2, 93. Conversely, the potential for stormier conditions (e.g., more hurricanes or typhoons) under GCC may increase mixing, disrupt oxygen stratification, and reduce oxygen depletions in some benthic environments 94. The complex interactions between GCC, hypoxia, and contaminants are not limited to nutrient and fertilizer runoff but are also influenced by interactions with other chemical contaminants, including PAHs, dioxins, and PCBs. It is predicted that GCC will increase environmental levels of PAHs and dioxins through biomass burning and may alter and remobilize legacy persistent organic pollutants such as PCBs 10, 95.

Hypoxia alone has been shown to act like an endocrine disruptor in aquatic species 96–101. Studies have also shown that hypoxia may increase the toxicity of exposures to PAHs, dioxins, and PCBs, leading to potential CITS 102, 103. Exposures to these chemical classes may also hinder the ability of species to respond to increased hypoxia under climate change (TICS scenario) 104. Interactions between hypoxia and contaminants demonstrate the complexity of direct and indirect parameters altered by GCC that could impair the health of aquatic organisms and populations.

While there is substantial evidence that hypoxia impairs the reproduction and development of fish 105, the mechanisms of toxicity on the endocrine system and HPG axis continue to be unclear. Laboratory and field studies have shown hypoxia-induced reductions in circulating levels of sex hormones (testosterone, 11-ketotestosterone, estradiol) and the egg yolk protein precursor vitellogenin concomitant with impaired reproduction and sex differentiation in fish 97, 100, 101, 106. Mechanisms of action that have been demonstrated to play roles in the hypoxia-induced reproductive impairments include altered expression of genes and impaired activity of enzymes responsible for steroid biosynthesis 98, 100, downregulation of the serotonergic pathway 107, and nongenomic actions of progestins on oocyte and sperm plasma membranes 108.

In addition to hypoxia alone, GCC-linked increases of some pollutants (e.g., PAHs, dioxins) and remobilization of others (e.g., PCBs) coupled with worsening hypoxia may impair the ability of organisms to respond to these dual environmental stressors. Exposures to contaminants such as PCDDs, PCDFs, planar PCBs, and PAHs elicit biological responses that are triggered by binding to the aryl hydrocarbon receptor (ahR). Functioning of the ahR pathway and its underlying mechanisms have been well described in vertebrates and include adaptive signaling upregulating xenobiotic metabolizing enzymes; toxic signaling causing adverse effects from high-affinity ligands, notably TCDD; and developmental signaling contributing to normal development of some tissues and organs 109.

On activation, ahR proteins form heterodimers with ahR nucleotranslocator proteins. The formation of these ahR/ahR nucleotranslocator complexes is regulated by ligand (or xenobiotic) binding. This complex can then bind to dioxin response elements upstream of gene coding regions, leading to altered gene expression 109. The ahR nucleotranslocator may also dimerize with the α-class of hypoxia-inducible factors (HIF-1α, HIF-2α, HIF-3α) to alter gene expression in response to low oxygen stress, leading to a cascade of physiological responses to low DO. Thus, because the ahR nucleotranslocator is shared across multiple signaling pathways, it is possible that activation of one pathway could inhibit activation of another pathway that depends on the ahR nucleotranslocator 110. While mechanisms of response to hypoxia and TCDD-like compounds are each fairly well understood, the crosstalk between these two pathways and mechanisms by which one pathway interferes with the other continue to be poorly understood 102, 103, 105, 110.

Studies in zebrafish larvae and cell cultures suggest that hypoxia may reduce ahR signaling and inhibit 2,3,7,8-TCDD induction of CYP1A (i.e., CITS pathway) 103, 110. However, these studies suggest that reciprocal crosstalk may not be occurring in that dioxins have not been observed to inhibit hypoxia signaling pathways (i.e., TICS pathway). A few studies have also examined the crosstalk between hypoxia and PAHs. Synergistic increases in teratogenicity have been previously demonstrated in fish exposed to mixtures of PAH-CYP1A inhibitors (e.g., fluoranthene) and PAH-ahR agonists (e.g., benzo[*a*]pyrene). Consistent with 2,3,7,8-TCDD/hypoxia studies, PAH-ahR agonist exposures in the presence of hypoxia reduced CYP1A activity in zebrafish embryos but did not increase teratogenicity 102. However, zebrafish coexposures to PAH-CYP1A inhibitors fluoranthene and α-napthaflavone and hypoxia were shown to cause teratogenicity by an unknown mechanism. Another interaction that has been explored with fish is an antagonistic interaction whereby PCB exposures make fish less tolerant to hypoxia (i.e., TICS pathway) by impeding the production and activity of glycolytic enzymes that are responsible for cellular energy production when oxygen is limited 104.

The situation presented by hypoxia serves as an example of the complexity of interactions between changing environmental conditions under GCC and the array of interactions of chemical and nonchemical stressors on biota. This case demonstrates that worsening hypoxia coupled with increasing pollution under GCC could act at multiple levels of biological organization and by multiple toxicological pathways, leading importantly to impaired reproduction and development of fish. Hypoxia itself can impair the HPG system, potentially eliciting impacts on reproduction and development, and TCDD-like contaminants could contribute to these impacts, albeit by very different mechanisms. A prospective AOP would be greatly beneficial in examining these complex interactions because it could help to identify and translate the range of interconnected mechanisms by which hypoxia and pollutants might interact to culminate in potential impacts on reproduction and development. This type of AOP construct could then be used to identify natural populations that might be subjected to these complex interactions for further risk analysis. It could also help to identify data gaps and other chemical contaminants that might act by similar pathways. To this end, a fish vitellogenin model has been used successfully in the field to identify mechanisms underlying hypoxia-induced endocrine disruption 99. This sort of modeling holds promise as a viable retrospective approach to determine the role of climate-induced hypoxia and nutrient runoff in fish HPG impairment. These types of retrospective analyses could be used as tools to identify populations projected to be susceptible to GCC increases in rainfall, hypoxia, and contaminant exposures.

## POLAR BEARS, GCC, AND ORGANOHALOGEN CONTAMINANTS

At the highest levels of biological organization, chemical and GCC exposures can influence the interactive dynamics of individuals, populations, and communities. For example, modifications of food-web dynamics by climate change can produce community-level changes that can impact how individual animals are exposed to contaminants. Prospective AOP analyses can help to predict and interpret potential ramifications of these complex GCC and pollutant interactions observed in the field.

As previously described, warming air and sea temperatures in Arctic and subarctic regions are among the strongest and best-documented climate change indicators 93, with resulting changes in the timing and extent of seasonal sea ice breakup having a profound influence on associated ecosystems 111, 112. Polar bears (*Ursus maritimus*) in the western Hudson Bay (WHB) region of Canada occupy the extreme southern range for this species, where GCC effects have been strongest. Dependent on the Hudson Bay sea ice for movement, mating, and feeding, WHB polar bears have suffered declining body condition, birth rates, and survival rates over the past 20 years. Measured declines have been concurrent with sea-ice breakup occurring three weeks earlier in the summer 113, 114. These changes appear to be influencing concentrations and profiles of chemical contaminants in WHB polar bears as well as the potential for resulting health effects. Moreover, polar bear decline may serve as a bioindicator of the overall declining health of these northerly ecosystems due to the combined impacts of GCC with other perturbations, including chemical exposures.

Polar bears feed on seals that inhabit both pack ice and open water. Fatty acid dietary tracers from polar bear adipose tissues demonstrate that males feed on larger pack ice–inhabiting bearded seals (*Erignathus barbatus*) and smaller ringed seals (*Pusa hispida*). Smaller female bears feed primarily on ringed seals 115, 116. Since the mid-1990s, the overall proportion of bearded seals in the WHB polar bear diet has decreased significantly, being replaced by more ringed seals as well as harp and harbor seals (*Phoca groenlandica* and *Phoca vitulina*, respectively) 115.

Concurrent with this dietary shift has been a change in the composition and concentrations of organohalogen (OHC) contaminants in polar bear adipose tissue. Concentrations of total PBDEs and β-hexachlorocyclohexane increased, as did DDT and DDT metabolite clearance, from 1991 through 2007 117. When corrected for dietary shifts during this time, noted accumulation and clearance rates were significantly higher. Changes in diet and contaminants have occurred simultaneously with earlier sea-ice breakup, though direct correlation has been difficult to develop because of the complexity and variability of dietary food webs, contaminant sources, and climate trends. Investigators continue to work on understanding how GCC is affecting the polar bear diet and the interactive mechanisms involved in increasing contaminant bioaccumulation and clearance rates 118. In a prospective scenario, it could be suggested that GCC-induced community-level perturbations are changing contaminant exposure patterns in WHB polar bears (i.e., CITS scenario).

During ice-free periods, when polar bears are forced ashore and prey are scarce, fasting leads to substantial body fat mobilization and body mass loss in polar bears, with WHB females fasting up to eight months through the denning and early cub-rearing periods 114, 119. In a study of OHC disposition in polar bears over an onshore 60-d (mean) fasting period, adipose, plasma, and milk concentrations of these compounds increased significantly with loss of body mass and body fat. Total chlordane-like chemicals and summed PCBs rose to near double their prefast concentrations, while DDT and DDT metabolites decreased 120. Increased milk concentrations of chlordanes and PCBs led to increases in body burdens of nursing cubs of the year. In pregnant WHB female polar bears, the actual “reproductive fast” averaged 192 d (*n* = 8) from the time the bears came ashore to emergence from their dens with new cubs, with average body mass loss of 43% of their prefast body weight 119. The actual increase in systemic and milk organochlorine concentrations that occurs through the reproductive fast is likely much greater than the near doubling documented over a 60-d portion of that fasting period. As the spring ice breakup comes earlier, feeding time on the pack ice for polar bears decreases and the effort they expend to return to shore takes a greater toll on their fat reserves. Females thus enter the reproductive fast with lower body fat stores, likely leading to greater concentrations of these contaminants in both maternal and young bears. Thus, a second stress on polar bears from sea-ice breakup is shorter hunting time on the ice and longer fasting periods, with both leading to greater mobilization of adipose-sequestered OHCs, an ADME effect that leads to substantially higher circulating and tissue body burdens of OHCs.

The seasonal increases of OHCs in fasting polar bears are notable as they bring systemic concentrations to levels similar to those of the more contaminated polar bears of the Svalbard Islands (Norway) and eastern Greenland 121. Of particular concern are cubs of WHB polar bears, due to the increased sensitivity of developing mammals to endocrine and immune system effects associated with these persistent halogenated organics. The OHC contaminants of the types and concentrations found in WHB polar bears have been correlated in polar bears with impaired thyroid hormone regulation 122, elevated progesterone 123 and depressed testosterone concentrations 124, and impaired humoral and cellular immune function 125, 126. Surrogate studies using arctic foxes and sled dogs fed minke whale blubber rich in OHCs or control pork-fat diets are helping to identify underlying mechanisms and cause-and-effect evidence for these correlative findings as dosing studies of polar bears are not feasible (reviewed in Letcher et al. 121).

In the present case study, effects of climate in elevating OHC concentrations (CITS) could lead to a TICS scenario due to resulting endocrine, immune, and neurodevelopment perturbations that could potentially hinder acclimatization to GCC stressors. The use of a prospective AOP approach allows for prediction of potential cause-and-effect relationships resulting from observed field data. These predictions can be used by risk assessors in the interpretation of existing findings in polar bears and in predicting the nature and magnitude of anticipated effects in more northerly populations of polar bears, where sea-ice retreat has not yet reached the extremes that confront the most southern WHB populations. In this complex case study where CITS leads to TICS, we also see the role that AOPs can play in integrating community-level GCC perturbations 127. Changes in food webs can lead to altered contaminant burdens in apex predators. The impaired health effects that could accompany these changes are expressed at the individual organism level, though in scenarios such as that in WHB polar bears, the accumulative effects on reproduction, recruitment, and even survival might lead to adverse population-level effects and potentially extirpation of some local populations.

## NESTLING PASSERINES, MERCURY, AND EXTREME CLIMATE EFFECTS

In addition to building prospective and theoretical AOP scenarios based on empirical findings in the fields of climate change and ecotoxicology, risk and resource injury assessors have recently been confronted with adverse outcomes in wild populations without straightforward explanatory pathways. When this occurs, working backward from adverse outcomes through mechanistic pathways can help to formulate hypotheses to identify causative factors (and discard unlikely scenarios). Development of cause-and-effect relationships that explain GCC–toxicant interactions in a specific population can provide models and hypotheses that can be applied to other populations faced with similar combinations of stressors.

Field studies have recently illustrated how ambient temperature and a contaminant interact as costressors on reproduction in tree swallows (*Tachycineta bicolor*). Following a report that mercury had reduced production of tree swallow nestlings during a drought but not during the previous year of normal weather 128, researchers established a large swallow population on mercury-contaminated and reference sites and tested for interactions between the effects of weather and mercury on swallow reproduction 129. Temperature and precipitation data from specific 5- to 10-d periods of the nesting cycle (egg production, incubation, featherless nestlings, and feathered nestlings) were examined separately to identify toxicity mechanisms for any observed effects. Earlier investigations had demonstrated elevated prey item and maternal and nestling blood mercury concentrations on the contaminated sites and weak but significant negative relationships between reproduction measures (egg-hatching success and survival of nestlings) and maternal blood mercury concentrations 128. As in previous years, nesting on the mercury-contaminated site reduced the production of young that survived to leave the nest (fledglings) by approximately 20%.

During the late nestling period, a stage when nestlings are able to thermoregulate effectively and parental behavior consists almost entirely of feeding nestlings and hunting, the relationship between maximum daily temperature and fledgling production was positive for swallows nesting on both types of site. This was expected because the emergence and abundance of insects often increase with temperature 130, so the likelihood of starvation is reduced regardless of contamination status. The more interesting finding was that during the early nestling period, when parents devote a substantial amount of time to managing the temperature of the nest (brooding) and less effort feeding their blind and featherless nestlings, there was a significant interaction between mercury and maximum daily temperature. Whereas higher temperatures during the early nestling period were related to higher fledgling production on reference sites, these early temperature elevations on mercury-contaminated sites resulted in significantly reduced fledgling production 129.

These findings explain the earlier observation that mercury impacted reproduction more during hot, dry conditions. As one of the first examples of its kind from free-living vertebrates, it underscores the importance of considering the interaction of climatological and contaminant costressors. The toxicity of unseasonable heat combined with mercury contamination is greater than existing mercury data predict, suggesting that the increasing frequency of extreme temperature events accompanying GCC 131 will lead to adverse effects where current exposures do not produce detectable impacts. What is unclear is whether the effects of this stressor combination are due to extreme weather modifying mercury toxicity (CITS) or mercury impacting the birds' ability to acclimate to extreme elevations in temperature (TICS). A mechanistic understanding of this chemical–climate interaction is necessary to fully anticipate its repercussions in the current scenario as well as predict the implications for other species and toxicants.

**Fig. 4:**
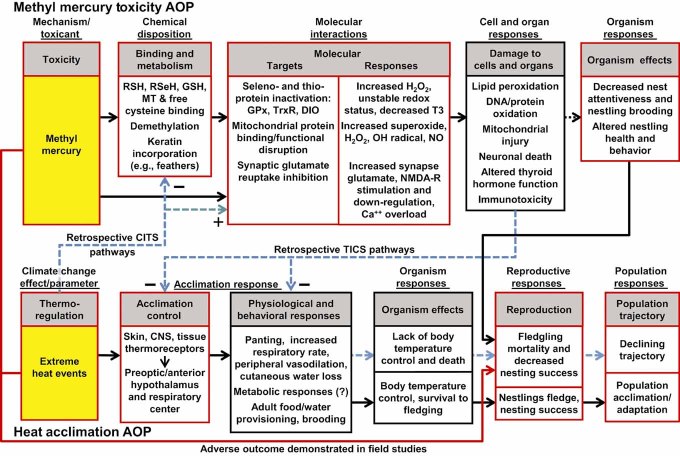
Adverse outcome pathways (AOPs) for investigating demonstrated 129 combined effects of extreme heat events and mercury toxicity in tree swallow nestlings. Retrospective pathways for potential climate-induced toxicant sensitivity (CITS) and toxicant-induced climate sensitivity (TICS) mechanisms are indicated. See Figure 2 for key to interactions. DIO = iodothyronine deiodinase; GPx = glutathione peroxidase; GSH = reduced glutathione; H_2_O_2_ = hydrogen peroxide; MT = metallothionein; NMDA-R = *N*-methyl-**d**-aspartic acid receptor; NO = nitric oxide; OH = hydroxyl; RSH = protein thiol; RSeH = protein selenol; T3 = triiodothyronine; TrxR = thioredoxine reductase. [Color figure can be seen in the online version of this article, available at http://wileyonlinelibrary.com.]

An AOP for this scenario requires a toxicity pathway for mercury (primarily methyl mercury [MeHg] in the study birds) and a pathway for acclimation to elevated heat conditions (hyperthermia) in tree swallow nestlings. An AOP was constructed that provides a starting point for a retrospective analysis of causation in the combinatory toxicity of weather and mercury in tree swallows (Fig. 4).

Work with live animals and in vitro studies provide insights into the mechanisms of MeHg toxicity (for review, see Farina et al. 132, 133). Once contaminated food is ingested, MeHg is absorbed effectively (>90%) from the gut and enters tissues as a cysteine-bound conjugate that mimics the amino acid methionine, moving freely into cells via amino acid transport proteins on cell membranes. Within the cell, the MeHg–cysteine bond is sufficiently labile to allow exchange reactions where MeHg is transferred to other, more reactive protein thiols (R-SH) and selenols (R-SeH), disrupting existing molecular structures and interfering with protein function. The MeHg inactivates or modifies enzymes active in a variety of mechanisms critical to cellular function. These include antioxidant defenses, such as glutathione peroxidase and thioredoxin reductase, which protect against reactive oxygen species (ROS). Interference with glutamate reuptake into astrocytes by MeHg leads to glutamate buildup in the synapse and overstimulation of *N*-methyl-**d**-aspartic acid receptor calcium channels on adjacent neurons. This and other ROS-generated membrane-disruption mechanisms lead to flooding of calcium into sensitive neuronal cell structures, where it disrupts mitochondria and causes neuronal cell death. The ramifications of these effects, in addition to direct interference of MeHg with mitochondrial oxidative phosphorylation components, are an increase in cellular occurrence of ROS leading to lipid peroxidation, DNA and protein oxidation, mitochondrial injury, and cell death. In the avian nervous system, such effects may lead to behavioral dysfunction, manifest in decreased/lethargic foraging effort and reduced incubation times in nesting adult birds 134. In nestlings, motor impairment predominates, with decreased exploratory movements and increased anomalous movements, consistent with observed reductions in the number and density of Purkinje cells in the cerebellum 135. The outcome of this mechanistic pathway could be death for nestlings should adult care or their own aberrant behavior (e.g., lack of vocalizing for food) lead to lack of adult provisioning.

Acclimation to elevated temperatures, or hyperthermia, in nestlings occurs primarily by means inherent to the nestling, dissipating heat (thermolysis) with little input from adult birds (reviewed in Dawson and Whittow 136). Peripheral and central neuronal and deep-body thermoreceptor inputs are integrated in the preoptic/anterior hypothalamus. A panting response generated in the respiratory center triggers open-mouth breathing with an elevated respiratory rate, leading to evaporative cooling. Peripheral vasodilation and cutaneous water loss from nonkeratinized skin also further cool the nestling. Continued adult provisioning under these conditions is important to maintain nestling hydration as evaporative cooling leads to body water loss. Should the nestling fail to maintain these behaviors, adults fail to provision, or elevated temperatures overwhelm thermolysis mechanisms, the nestling will die.

Examining the details of this case retrospectively allows isolation of some mechanisms as being more likely than others. First, it is notable that the mercury–temperature interaction occurred only during the first week of the nestling's life, not during egg formation or incubation or in older nestlings. Parental care during the early nestling stage involves feeding and brooding, either of which could be sensitive to mercury and temperature. However, if food availability or parental foraging ability were the target of the interaction, one would expect an even stronger interaction in late-stage nestlings that require ever greater amounts of food. The lack of effects in late-stage nestlings, as well as during incubation, suggests that a deficit in parental care of the youngest nestlings was not a causative factor in their reduced fledging rates. An additional parental behavior whose alteration might have affected nestling survival was male provisioning of feathers during nest construction, such that nests on mercury-contaminated sites were more insulated, leading to mortality during hot weather. However, the number and mass of feathers in nests did not differ between mercury and reference sites (D. Cristol, unpublished data).

In the absence of parental effects on nestling survival, a retrospective assessment of CITS and TICS pathways within the AOP provides insight into potential interactive mechanisms. Were extreme heat events affecting MeHg toxicity (CITS), it is likely they would impact either the disposition of MeHg or its interaction with molecular targets (Fig. 4). Young nestling body temperatures, not fully capable of thermolysis, rise with ambient temperatures, which could affect the distribution, binding, and sequestration of systemic MeHg. For example, nestling thyroid hormone levels on mercury sites, with already depressed 3,3′,5-triiodothyronine concentrations 137, could be driven down further due to increased MeHg binding of iodothyronine deiodinase, the selenoprotein responsible for the conversion of thyroxine to 3,3′,5-triiodothyronine. Hyperthermic conditions, which increase ROS generation in birds under uncontaminated conditions 138, could increase MeHg-induced ROS generation and the subsequent oxidative stress in the nestling. This effect could be additive or even synergistic, due to interaction of multiple mechanisms of ROS generation. Climate impacts on MeHg toxicity are thus plausible and, based on scenarios generated from the AOP, testable to determine their validity.

The alternative pathway, where toxic effects of MeHg interfere with nestling acclimation to hyperthermic conditions (TICS), is also plausible as developing nestlings are particularly sensitive to MeHg toxicity 139, 140. The limited thermolytic mechanisms available to cavity-inhabiting nestlings proceed through neurological pathways that could be lost due to neurotoxic effects of MeHg, impacting neuronal thermoreceptors, central control centers, or efferent neurons that extend to control peripheral responses. Cessation of panting, decreases in respiration rate, or loss of peripheral vasodilation could quickly lead to lethal overheating in small, immobile nestlings. Alternatively, aberrant nestling behavior during parental feeding bouts, such as lack of or inappropriate begging activities, could lead to ineffective nestling provisioning and decreased food and water consumption that would also prove lethal during periods when evaporative water loss is a primary thermolysis mechanism. As with the retrospective CITS scenarios, the TICS mechanisms are plausible and testable in the field or laboratory.

A fruitful next step in unraveling the mechanism of mercury–GCC interactions would be a mercury dosing study varying temperature and MeHg treatments to examine the effects of mercury and temperature on nestling bird survival to one week of age. Testing of TICS mechanisms might prove expeditious as thermolytic behaviors such as gaping mouth, panting, and elevated respiratory rates, or the lack thereof due to MeHg, would provide easily accessible evidence of hindered acclimation mechanisms. Alternatively, increased ROS occurrence in thermally stressed and MeHg-dosed nestlings would be evidence that temperature was accelerating the ROS generation pathways. It would not be out of the question if both types of effects were observed, with a temporal progression of heat-induced increases of ROS generation leading to greater neurodegeneration and a loss of thermolytic capacity. The AOP in Figure 4 incorporates mechanisms from across many species but is a starting place for prospective analyses. Fine-tuning the components by developing data for passerine species would provide more accurate predictions for tree swallows. Understanding how species differences modify the pathways would increase the breadth of the model's applicability.

Whichever pathway or combination of pathways is responsible for climate–stressor interactions in the tree swallows, the ramifications are important for conservation and species protection. If mercury impairs the developing thermoregulatory system, leading to higher mortality of young nestlings during unseasonable heat, and the identified mechanism is conserved across bird species, risk managers would have a strong basis for predicting the effects of GCC on temperate songbirds with predicted exposure to mercury. If, instead, temperature exacerbated MeHg-induced ROS generation, neurotoxicity, or other toxic responses, there could be wide repercussions for the range of other compounds that work via similar mechanisms of toxic action (e.g., other cationic metals, compounds with electrophilic reactive metabolites). In either case, if mitigation or habitat triage were called for, reducing environmental mercury concentrations and/or availability would be, in the long run, the most important approaches, with conservation resources applied selectively to populations residing at the edge of their physiological temperature tolerance range.

## CONCLUSION

These case studies demonstrate, at a mechanistic level, how GCC can interact with known toxic chemicals to change anticipated exposure–effect outcomes. In the process, they illustrate essential concepts for understanding the combined stresses of GCC and chemical contaminants. What stands out as an important lesson is that environmental toxicologists cannot ignore the increasing influences of climate change when developing effects inputs for chemical and contaminant assessments. It would be equally unwise to forecast the effects of GCC without considering the interaction between climatic variables and contaminants that can alter current risk outcomes. This is not to suggest that all chemical interactions with all species will be altered by GCC, but rather that, in risk and injury assessment scenarios, careful consideration should be given as to whether inclusion of GCC as a costressor is necessary to avoid erroneous estimates of impacts in study systems or defined populations 141.

Recognizing the importance of potential climate–chemical interactions in assessing risk is the crucial first step, and a path forward using AOPs is suggested. Through AOPs, the stepwise progression from exposure to chemical–receptor interaction to molecular, cellular, tissue, organ, and organismal responses can be tracked. The influence of changing climate variables can be tested, extrapolated, or predicted at critical points along the AOP, providing data on CITS. Similarly, an understanding of biological processes necessary for acclimation to GCC effects allows anticipation of how chemicals might perturb the acclimation process, leading to an understanding of TICS. As toxicity mechanisms are often shared by wide chemical classes and climate acclimation mechanisms are shared across taxonomically similar organisms, established climate–chemical AOPs provide a means by which important climate–chemical interactions can be characterized and applied across broad chemical classes and species groups of concern.

Building on pathway approaches, the case studies demonstrate the significance of the timing, duration, and overlap of exposures to chemical and climate stressors. Of similar importance is the recognition that sublethal alterations due to acclimation to the dual stresses of GCC and contaminant exposures can negatively impact an organism's fitness and survival. To answer questions of how climate and chemicals might interact as costressors, prospective scenarios can be built based on known or theoretical AOPs that incorporate climate modifications or, alternatively, on climate acclimation or adaptation processes that incorporate toxicological modifications. Conversely, when adverse outcomes with suspected climate and chemical interactions are observed, a retrospective analysis can be used to identify knowledge gaps and propose and test hypotheses that might identify the mechanistic basis for the interaction. Once clarified, the findings from both prospective and retrospective assessments can be used to identify and improve our ability to predict impacts occurring in the field.

Although the case studies addressed nonhuman impact assessments, the methods described will work equally well in GCC–chemical interaction scenarios with humans. Increases in human mortality from cardiovascular system– and respiratory system–related complications during heat waves are well established, suggesting one might work retrospectively through the physiological processes involved to identify the knowledge gaps and propose hypotheses and experiments that may explain the role of chemicals in morbidity and mortality due to combined temperature and contaminant exposures. Although extensive research along these lines has not been carried out, heat waves will clearly be a more frequent event as the result of GCC, and assessing risk of human deaths in major cities is going to be an important part of the planning and risk-assessment processes 142. Increased formation of secondary photochemical products such as ground-level ozone, which also has a significant association with increased morbidity and mortality 143, a rise in the number of wildfires, and increasing desertification of many regions in the world will further add to the burden of increasing atmospheric particulates, heightened cardiopulmonary complications, and compromised global human health. Understanding the mechanism for these deaths will require identification of the independent and interactive modes of action of temperature, ozone, and particulate matter. Once their respective roles are elucidated through both retrospective and prospective mechanistic investigations, the relative benefits of mitigative measures (e.g., reducing particulate matter and ozone precursors) can be assessed.

What is clear from the examples provided here is that a better understanding of how toxicological mechanisms interact with climate-induced stressors will provide a solid platform for improved effect assessments for both humans and wildlife. In chemical risk assessments for human health, retrospective AOP approaches can provide the biological plausibility that will inform predictions of changed patterns of disease as a result of GCC. Epidemiologists can use empirical data from existing and past associations between disease and climate to make projections about the human health implications of GCC, tying them to toxicological mechanisms through AOP scenarios. For environmental managers, AOP constructs and toxicological mechanisms allow prediction of GCC–contaminant interactions, which can facilitate refocusing and prioritization of regulatory efforts, species protection plans, research agendas, and conservation planning.
